# Clinical experience of noninvasive prenatal testing for rare chromosome abnormalities in singleton pregnancies

**DOI:** 10.3389/fgene.2022.955694

**Published:** 2022-09-26

**Authors:** Ting Hu, Jiamin Wang, Qian Zhu, Zhu Zhang, Rui Hu, Like Xiao, Yunyuan Yang, Na Liao, Sha Liu, He Wang, Xiaoyu Niu, Shanling Liu

**Affiliations:** ^1^ Department of Medical Genetics, West China Second University Hospital, Sichuan University, Chengdu, Sichuan, China; ^2^ Department of Obstetrics and Gynecology, West China Second University Hospital, Sichuan University, Chengdu, Sichuan, China; ^3^ Key Laboratory of Birth Defects and Related Diseases of Women and Children (Sichuan University), Ministry of Education, Chengdu, China

**Keywords:** NIPS for rare chromosome abnormalities noninvasive prenatal screening, rare chromosomal abnormality, positive predictive value, chromosomal microarray analysis, prenatal diagnosis

## Abstract

**Objectives:** The study aimed to investigate the clinical use of noninvasive prenatal testing (NIPT) for common fetal aneuploidies as a prenatal screening tool for the detection of rare chromosomal abnormalities (RCAs).

**Methods:** Gravidas with positive NIPT results for RCAs who subsequently underwent amniocentesis for a single nucleotide polymorphism array (SNP array) were recruited. The degrees of concordance between the NIPT and SNP array were classified into full concordance, partial concordance, and discordance. The positive predictive value (PPV) was used to evaluate the performance of NIPT.

**Results:** The screen-positivity rate of NIPT for RCAs was 0.5% (842/158,824). Of the 528 gravidas who underwent amniocentesis, 29.2% (154/528) were confirmed to have positive prenatal SNP array results. PPVs for rare autosomal trisomies (RATs) and segmental imbalances were 6.1% (7/115) and 21.1% (87/413), respectively. Regions of homozygosity/uniparental disomy (ROH/UPD) were identified in 9.5% (50/528) of gravidas. The PPV for clinically significant findings was 8.0% (42/528), including 7 cases with mosaic RATs, 30 with pathogenic/likely pathogenic copy number variants, and 5 with imprinting disorders.

**Conclusion:** NIPT for common fetal aneuploidies yielded low PPVs for RATs, moderate PPVs for segmental imbalances, and incidental findings for ROH/UPD. Due to the low PPV for clinically significant findings, NIPT for common fetal aneuploidies need to be noticed for RCAs.

## Introduction

Noninvasive prenatal testing (NIPT), also known as cell-free fetal DNA (cff-DNA) testing, is mainly based on massive parallel sequencing (MPS), and it has been used to screen for common fetal aneuploidies in more than 60 countries since 2011 (Palomaki et al., 2011). NIPT is highly sensitive and specific for the detection of trisomies 13, 18, and 21 (Committee on Genetics Society for Maternal for Maternal-Fetal Medicine, 2015; Taylor-Phillips et al., 2016), which has led to a reduction in invasive diagnostic testing requests by up to 40% to avoid procedure-related miscarriage risks (Wong and Lo, 2016). Recently, rare autosomal trisomies, well-known microdeletion/microduplication syndromes (MMSs), and genome-wide copy number variants (CNVs) have been added by some laboratories as expanded screening items (Wapner et al., 2012; Wapner et al., 2015; Benn and Grati, 2018). Apoptotic placental cells from the cytotrophoblast mixed with maternal cell-free DNA are the primary sources of cff-DNA in maternal circulation (Tjoa et al., 2006; Faas et al., 2012); hence, factors like confined placental mosaicism (CPM) and maternal genomic contributions affect the accuracy of NIPT results (Bianchi and Wilkins-Haug, 2014). Thus, all positive NIPT results should be confirmed by invasive diagnostic testing (Mardy and Wapner, 2016; Cherry et al., 2017).

Chromosomal microarray analysis (CMA), a high-resolution genomic technology used to detect CNVs, has been recommended as a first-tier test for the postnatal evaluation of individuals with unexplained developmental delay, intellectual disability, autism spectrum disorders, or multiple congenital anomalies, including prenatal evaluation of fetuses with structural anomalies observed by ultrasound (Manning et al., 2010; Miller et al., 2010; American College of Obstetricians and Gynecologists Committee on Genetics, 2013). Furthermore, single-nucleotide polymorphism (SNP) arrays can additionally identify haploidy, triploidy, and regions of homozygosity (ROH) (Levy et al., 2014). The pathogenesis of ROHs includes imprinting effects caused by uniparental disomy (UPD) (Robinson, 2000) and increased susceptibility to complex diseases caused by homozygous mutations in autosomal recessive genes (Campbell et al., 2007; Ku et al., 2011).

The utility of NIPT for specific MMSs with moderate to high positive predictive values (PPVs), including DiGeorge syndrome (DGS), Prader–Willi/Angleman syndrome (PWS/AS), cri du chat (CDC), and 1p36 microdeletion (1p36 del) syndrome, has been shown (Dar et al., 2014; Gross et al., 2016; Petersen et al., 2017; Liang et al., 2019). However, there is still a paucity of research focusing on rare chromosomal abnormalities (RCAs) detected by NIPT for common fetal aneuploidies. Therefore, in this retrospective cohort study of 158,919 singleton pregnancies, we evaluated the clinical use of the NIPT as a prenatal screening tool for the detection of RCAs, including aneuploidies and segmental imbalances.

## Materials and methods

### Patients

For this study, singleton pregnancies at a tertiary-level referral center (West China Second University Hospital, Sichuan University) were included from January 2016 to December 2020. Trained clinical geneticists performed pretest counseling. Before an NIPT or SNP array analysis, we obtained written informed consent from all gravidas who agreed to undergo NIPT or consecutive amniocentesis due to positive NIPT results. The Medical Ethics Committee of the West China Second University Hospital approved the study.

For NIPT, the inclusion criteria were as follows: 1) patients with advanced maternal age ≥35 years who declined invasive procedures; 2) patients with high risk for first- or second-trimester maternal serum screening (T21 ≥ 1/270, T18 ≥ 1/350) who declined invasive procedures; 3) intermediate risk for maternal serum screening (T21:1/270–1/1,000, T18:1/350–1/1,000); 4) fetuses with soft markers detected by ultrasound, including nuchal translucency of 2.5–3.0 mm; 5) positive family history, such as any affected offspring with Down syndrome; and 6) pregnant women who prefer NIPT to maternal serum screening without any clinical indications. The exclusion criteria, according to the current standard practice in China, were as follows: 1) pregnancy gestation period <12 weeks; 2) fetal structural anomalies detected by ultrasound before NIPT; 3) pregnant women with chromosomal abnormalities; 4) multiple pregnancies or co-twin demise after 12 weeks; 5) pregnant women who received stem cell therapy, transplant surgery, allogeneic blood products, or immunotherapy within one year; and 6) pregnant women with malignant tumors. Blood samples (10 ml) from the gravidas were collected in cell-free DNA BCT tubes (Streck, Omaha, United States).

All gravidas with positive NIPT results for RCAs, including rare autosomal trisomies (RATs) and segmental imbalances, were advised to undergo amniocentesis for SNP analysis after 16 gestational weeks. To assess the clinical use of NIPT for RCAs, the exclusion criteria were as follows: 1) positive NIPT results for common trisomies (T21/T18/T13); 2) positive NIPT results for sex chromosome aneuploidies (SCAs); and 3) pregnant women who declined amniocentesis or who underwent amniocentesis for traditional cytogenetics (e.g., karyotype alone) but declined SNP analysis. Fetal samples (20 ml) were obtained through amniocentesis. Clear amniotic fluid samples were tested directly, while blood-stained samples were cultured before the SNP array experiments. Additionally, peripheral blood samples of the parents were obtained to confirm that fetal CNVs were inherited or *de novo*, and ROHs were separated from UPDs.

### Noninvasive prenatal testing

Plasma from the blood samples was isolated within 24 h by two-step centrifugation. All procedures, including cell-free DNA extraction, purification, library construction, and quantification, were performed using the fetal chromosome aneuploidy (T21/T18/T13) test kit (Berry Genomics, Beijing, China). MPS was performed on the NextSeq CN500 platform (Berry Genomics, Beijing, China) with 36-bp single-end reads, resulting in 5 million total reads, which corresponds to a 0.05× sequencing depth. GC bias was eliminated by bioinformatic methods combined with local weighted polynomial regression. Raw reads were aligned to the human reference genome, GRCh37 (hg19). Each chromosome with an absolute Z-score greater than 3 was marked with chromosome aneuploidies. CNVs of ≥ 2 Mb were detected using the RUPA algorithm developed by Berry Genomics.

### Chromosomal microarray analysis

This procedure was described in our previous study (Hu et al., 2021). CNVs >100 kb or those that affected >50 contiguous probes and ROHs >10 Mb were considered. The pathogenicity of the detected CNVs was according to the criteria of the American College of Medical Genetics and Genomics (ACMG) and Clinical Genome Resource (ClinGen) Technical standards (Riggs et al., 2020). When the limit with which CMA can be expected to detect low-level mosaicism was 10%–20% (Cross et al., 2007; Scott et al., 2010; Hall et al., 2014), we simultaneously performed an interphase fluorescence *in situ* hybridization analysis (FISH) when CMA detected mosaicism (≥10%). When both a gain and loss of more than 5 Mb were detected in one fetal sample, peripheral blood samples of the parents were karyotyped to confirm whether the parents had chromosomal balanced translocations or inversions. The nomenclature of CMA and karyotypes is according to the International System for Human Genomic Nomenclature (ISCN) 2020 (McGowan-Jordan et al., 2020).

### Data analysis

The positive results of NIPT for rare RCAs were classified into two groups: 1) RATs and 2) segmental imbalances; however, the positive results of CMA were classified into three groups: 1) RATs (including mosaic aneuploidies), 2) segmental imbalances, and 3) ROH/UPDs. Clinically significant findings included RATs, pathogenic/likely pathogenic (P/LP) CNVs, and UPDs associated with imprinting disorders.

We compared the NIPT results with those of CMA and classified them into four categories: 1) full concordance: those with consistent aneuploidy results, or with consistent cytoband and copy number gain/loss between NIPT and CMA; 2) partial concordance: at least one of the findings was consistent, but additional findings were detected only by one platform (NIPT/CMA) (for example, NIPT was positive for 20p13p12.1 duplication, and CMA detected 20p13p12.1 duplication and 9p24.3p23 deletion); aneuploidies or segmental imbalances were detected by NIPT but ROH/UPD was confirmed by CMA; and 3) discordance: none of the findings detected by NIPT and CMA were consistent (for example, NIPT was positive for trisomy 6, and CMA was negative; or NIPT was positive for trisomy 7, and CMA detected 16p11.2 deletion).

### Clinical follow-up assessments

We performed clinical follow-up assessments 6 months to 3 years postpartum on all gravidas who underwent amniocentesis for SNP analysis. In addition, data on circumstances after birth, including the gestational age of delivery, birth weight, postnatal imaging, developmental details diagnosed by pediatricians, and perinatal or infant death, were collected. For fetuses treated with termination of pregnancy (TOP), the indications such as chromosomal aberrations, abnormalities of ultrasound findings, miscarriage following amniocentesis, premature delivery, and still births, were obtained through hospital information systems.

### Statistical analysis

A statistical analysis was performed by SPSS Statistics software v24.0 (IBM SPSS, Armonk, NY, United States). Continuous variables were compared by Student’s *t*-test, and categorical variables were compared by chi-squared or Fisher’s exact analysis, as appropriate. A *p*-value < 0.05 was considered to indicate statistical significance.

## Results

### Patient characteristics

**FIGURE 1:**
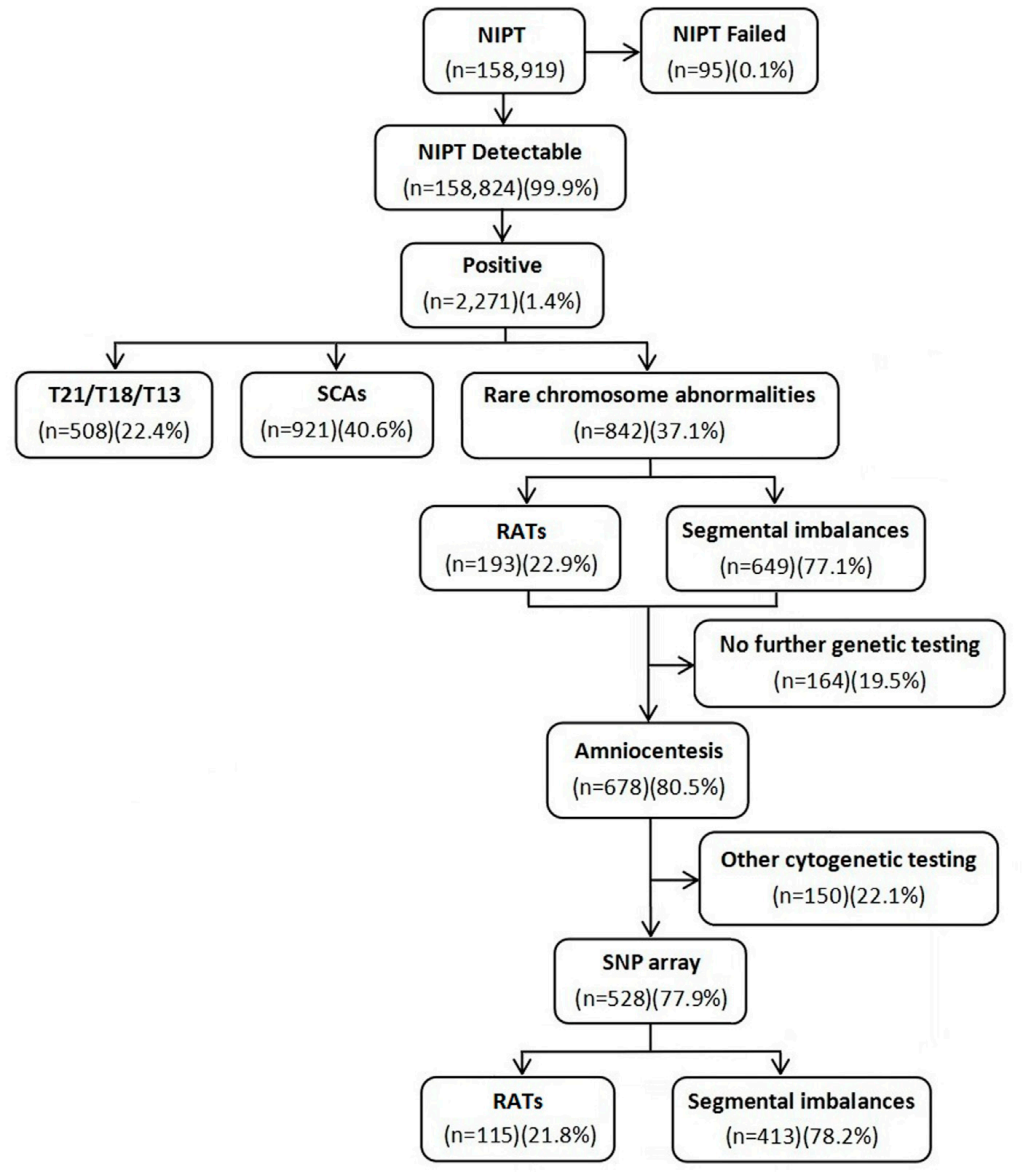
Flow diagram of the study.

A total of 158,919 gravidas were recruited to undergo NIPT in a 5-year retrospective study to evaluate the clinical value of NIPT for rare RCAs, including aneuploidies, segmental imbalances, and ROH/UPD. The test had a failure rate of 0.1% in 95 cases. A total of 508 (0.3%) cases with positive screen results for common trisomies (T21/T18/T13) and 921 (0.6%) cases for SCAs were excluded. In the 842 (0.5%) gravidas with positive screen results for RCAs, including 193 (22.9%) at high risk for RATs and 649 (77.1%) for segmental imbalances, 528 gravidas underwent consecutive amniocentesis for prenatal diagnosis by the SNP array. The flow diagram of the study is shown in Figure 1. The maternal age ranged from 17 to 44 years (29.1 ± 4.7 years), with 13.8% (73/528) being of advanced maternal age. The gestational age for NIPT and amniocentesis ranged from 12 to 27^+5^ weeks (19.7 ± 5.7 weeks) and 17 to 30^+1^ weeks (20.5 ± 3.3 weeks), respectively.

Of the 528 positive NIPT results, there were 115 fetuses at high risk for RATs, including 10 fetuses with multiple chromosomes, while the remaining 413 fetuses were at high risk for segmental imbalances, including 16 fetuses with multiple chromosomes. The SNP array was successfully performed in all gravidas; 154 (29.2%) cases had positive results, including 7 (4.5%, 7/154) fetuses with mosaic RATs, 97 (63.0%, 97/154) fetuses with segmental imbalances, and 50 (32.5%, 50/154) fetuses with UPD/ROH (Table 1). The concordance between the RCAs detected by NIPT and consecutive CMA results is shown in Table 2. No significant difference in maternal age was observed between the positive and negative SNP array groups (28.8 ± 5.1 years vs. 29.2 ± 4.5; *p* = 0.350).

**TABLE 1:** Summary of the CMA results of 528 fetuses with positive NIPT results.

Chromosome	NIPT (n)	CMA (n)				PPV (%, 95% CI)
		Rare aneuploidy	Segmental imbalance	ROH/UPD	Normal	
Chr 1	16	—	1	6	9	43.8, 16.6–71.1
Chr 2	28	—	5	6	17	39.3, 20.0–58.6
Chr 3	25	—	4	2	19	24.0, 6.0–42.0
Chr 4	15	—	2	4	9	40.0, 11.9–68.1
Chr 5	19	—	8 + 1^∆^	—	10	42.1, 17.7–66.6
Chr 6	8	—	1	6	1	87.5, 57.1–117.1
Chr 7	108	—	5 + 2^∆^	2	99	6.5, 1.8–11.2
Chr 8	48	—	8	5	35	27.1, 14.0–41.1
Chr 9	20	5^a^	2	3	10	50.0, 26.0–74.0
Chr 10	16	—	6	_	10	37.5, 10.9–69.1
Chr 11	19	—	3 + 1^∆^	2	13	26.3, 4.5–68.1
Chr 12	7	—	3	1	3	57.1, 7.7–106.6
Chr 13	14	—	5	1	8	42.9, 13.2–72.5
Chr 14	18	—	1 + 3^∆^	1	13	11.1, -5.0–27.2
Chr 15	20	1^a^	5 + 1^∆^	1	12	35.0, 12.1–57.9
Chr 16	33	1^a^	5	9	18	45.5, 27.5–63.4
Chr 17	7	—	1	1	5	28.6, -16.6–73.7
Chr 18	22	—	8 + 1^∆^	—	13	36.4, 14.5–58.2
Chr 19	1	—	—	—	1	—
Chr 20	26	—	3	—	23	11.5, -1.6–24.7
Chr 21	21	—	3	—	18	14.3, -2.0–30.6
Chr 22	11	—	6	—	5	54.5, 19.5–89.6
Multiple chromosome	26	—	2 + 1^∆^	—	23	7.7, -3.3–18.7
**Total (n)**	528	7	87 + 10^∆^	50	374	27.3, 23.5–31.1

a Mosaic aneuploidies; ∆ the positive results discordant with NIPT.

NIPT: noninvasive prenatal testing; CMA: chromosomal microarray analysis. ROH: regions of homozygosity; UPD: uniparental disomy; PPV, positive predictive value. CI, confidence interval.

**TABLE 2:** Concordance between the RCAs detected by NIPT and consecutive CMA results.

Chromosome	CMA (n)																			
	Rare aneuploidies	Segmental imbalance								ROH/UPD										
	Full concordance	Full concordance				Partial concordance				Discordance				Partial concordance						
														iUPD			hUPD			ROH
		mat	Pat	*de novo*	NA	mat	pat	*de novo*	NA	mat	pat	*de novo*	NA	mat	pat	NA	mat	pat	NA	
Chr 1	—	1	—	—	—	—	—	—	—	—	—	—	—	—	—	3	—	—	—	3
Chr 2	—	1	—	—	4	—	—	—	—	—	—	—	—	1	—	1	1	—	—	3
Chr 3	—	2	—	—	1	—	—	1	—	—	—	—	—	—	—	—	1	—	—	1
Chr 4	—	—	—	1	1	—	—	—	—	—	—	—	—	—	1	2	1	—	—	—
Chr 5	—	3	—	—	5	—	—	—	—	—	1	—	—	—	—	—	—	—	—	—
Chr 6	—	1	—	—	—	—	—	—	—	—	—	—	—	3	1	1	—	—	—	1
Chr 7	—	1	1	1	2	—	—	—	—	—	—	1	1	—	—	2	—	—	—	—
Chr 8	—	2	—	—	4	—	—	2	-	—	—	—	—	1	—	1	—	—	—	3
Chr 9	5^a^	—	—	—	—	1	—	—	1	—	—	—	—	—	—	1	—	1	—	1
Chr 10	—	3	—	—	2	—	—	—	—	—	—	—	—	—	—	—	—	—	—	—
Chr 11	—	2	—	1	—	—	—	—	—	1	—	—	—	—	—	—	1	1	—	—
Chr 12	—	—	1	—	1	—	—	1	—	—	—	—	—	—	—	—	—	—	—	1
Chr 13	—	3	—	—	2	—	—	—	—	—	—	—	—	—	—	—	—	—	—	1
Chr 14	—	1	—	—	—	—	—	—	—	1	—	—	2	—	—	—	1	—	—	—
Chr 15	1^a^	2	—	—	3	—	—	—	—	1	—	—	—	—	—	—	1	—	—	—
Chr 16	1^a^	1	—	—	4	—	—	—	—	—	—	—	—	—	—	—	3	—	—	6
Chr 17	—	1	—	—	—	—	—	1	—	—	—	—	—	—	—	1	—	—	—	—
Chr 18	—	1	—	—	4	2	—	—	1	—	—	—	1	—	—	—	—	—	—	—
Chr 19	—	—	—	—	—	—	—	—	—	—	—	—	—	—	—	—	—	—	—	—
Chr 20	—	—	—	—	1	—	—	2	-	—	—	—	—	—	—	—	—	—	—	—
Chr 21	—	2	—	1	—	—	—	—	—	—	—	—	—	—	—	—	—	—	—	—
Chr 22	—	1	—	3	3	—	—	—	—	—	—	—	—	—	—	—	—	—	—	—
Multiple chromosome	—	1	—	—	—	—	—	—	—	—	—	—	1	—	—	—	—	—	—	—
Total (n (%, 95% CI))	7^a^(6.1, 1.7–10.5)	29	2	7	37	3	—	7	2	3	1	1	5	5	2	12	9	2	—	20 (3.8, 2.2–5.4)
		75 (18.2, 14.4–21.9)				12 (2.9, 1.3–4.5)								19 (3.6, 2.0–5.2)			11 (2.1, 0.9–3.3)			
						87 (21.1, 17.1–25.0)^∆^											50 (9.5, 7.0–12.0)			

a Mosaic aneuploidies; ∆ the positive results discordant with NIPT.

RCAs, rare chromosome abnormalities; NIPT, noninvasive prenatal testing; CMA, chromosomal microarray analysis; NA, not available; ROH, regions of homozygosity; UPD, uniparental disomy; PPV, positive predictive value; CI, confidence intervals.

### Rare autosomal trisomies

The PPV for RATs was 6.1% (7/115, 95% confidence interval (CI), 1.7%–10.5%) (Table 2). The SNP array confirmed that all RATs were as mosaicism. The most common aneuploidy was mosaic trisomy 9, including five cases with mosaic proportions ranging from 15 % to 29%. The other two cases were mosaic trisomy 15 (26%) and mosaic trisomy 16 (13%). All the mosaic aneuploidies were simultaneously confirmed using FISH in amniotic fluids.

### Segmental imbalances

The PPV for segmental imbalances was 21.1 % (87/413, 95 % CI, 17.1%–25.0%). A total of 111 segmental imbalances in 97 cases were detected by the SNP array, comprising 75 (77.3%) cases with full concordance, 12 (12.4%) with partial concordance, and 10 (10.3%) with discordance. Approximately, 57.7% (64/111) of the CNVs were <5 Mb, 19.8% (22/111) ranged from 5 to 10 Mb, and 22.5% (25/111) were >10 Mb, with a concordance rate of 75.0% (48/64), 86.4% (19/22), and 88.0% (22/25) between the NIPT and SNP arrays, respectively (*p* = 0.276).

The PPV for clinically significant CNVs (18 P CNVs and 12 LP CNVs) was 7.3% (30/413; 95 % CI, 4.8%–9.8%). There were 38 clinically significant CNVs including 23 P CNVs and 15 LP CNVs detected by the SNP array in 36 cases, comprising 24 (66.7%) cases with full concordance, six (16.7%) with partial concordance and six (16.7%) with discordance. For the well-known MMSs confirmed by the SNP analysis, 1p36 deletion, 15q11q13 (PWS/AS) duplication, DGS, and 22q11.2 duplication were all detected by NIPT, while 50% (2/4) of the CDC cases were ignored. The details of segmental imbalances detected by the SNP array are shown in Supplementary Table S1.

Parental confirmation by the SNP array was performed in 54.6% (53/97) of cases, while fetuses were detected with segmental imbalances by CMA, including 35, 3, and 15 cases of maternal, paternal inheritance, and *de novo* inheritance, respectively (Table 2). The proportion of full concordance between NIPT and CMA in cases with maternally inherited CNVs was significantly higher than that in those with paternal-inherited or *de novo* CNVs (82.9% (29/35) vs. 50.0% (9/18); *p* = 0.012) (Table 2). In addition, of the 10 cases with subchromosomal unbalanced rearrangements, except for one couple who refused to perform karyotyping (No. 84), six cases had inherited parental balanced translocations (Nos. 78, 79, 81, 83, 86, and 87), one case inherited paternal pericentric inversion (No. 77), and two cases inherited a mother derivative chromosome associated with intellectual disability (Nos. 80 and 82) (Supplementary Table S1).

### Regions of homozygosity/uniparental disomy

We incidentally detected 50 (9.5 %) fetuses by the SNP array with ROH larger than 10 Mb restricted to one chromosome, all relatively consistent with the NIPT results. Chromosome 16 was the most frequently involved (nine cases), followed by chromosomes 1, 2, and 6 (six cases). None of the fetuses was from consanguineous couples.

In total, 30 cases were confirmed as UPD: 18 cases were diagnosed with isodisomy as ROHs detected by the SNP analysis involving the whole chromosome (including six cases with a confirmed parental source of ROHs), and 12 iso-heterodisomy cases were verified by parental blood samples. The most frequent UPD was UPD6 (five cases), followed by UPD4 (four cases), and UPD1, UPD2, and UPD16 (three cases).

There were 11 cases of ROHs associated with imprinted chromosomes. Except for three couples who refused to perform parental confirmations, imprinting disorders were confirmed in five of the eight cases, including transient neonatal diabetes mellitus (pUPD6), Silver–Russell syndrome (mUPD11), Beckwith–Wiedemann syndrome (pUPD11), Temple syndrome (mUPD14), and PWS (mUPD15). The details of ROHs detected by the SNP analysis are shown in Supplementary Table S2.

### Clinical follow-up assessments

Clinical follow-up results were obtained in 90.2% of cases (476/528). Except for the fetuses lost to follow-up, the rates of normal infants, termination of pregnancy (TOP), and birth with defects (including neonatal demises without physical birth defects) in those without chromosomal aberrations by the SNP array were 94.9% (314/331), 2.4% (8/331), and 2.7% (9/331), respectively, while in those with chromosomal aberrations by the SNP array were 47.5% (69/145), 49.0% (71/145), and 3.4% (5/145), respectively (Table 3).

**TABLE 3:** Clinical follow-up assessment of the 528 fetuses detected by CMA.

**SNP array**		**Total**	**Loss of follow-up**	**TOP**			**Birth**		
				**Chromosomal abnormality**	**Ultrasound abnormality**	**Other**	**Normal**	**Birth defect**	**Death after birth**
Rare aneuploidies (mosaic)		7	—	7	—	—	—	—	—
Segmental imbalances	P/LP CNVs	36	1	29	—	—	6	—	—
	VUS	61	4	7	1	—	—	—	—
ROH/UPD	UPD	30	1	14	7	1	5	2	—
	ROH	20	3	3	1	1	10	1	1
Normal		374	43	—	6	2	314	9	—
Total		528	52	60	15	4	383	12	2

CMA, chromosomal microarray analysis; SNP, single nucleotide polymorphism; TOP, termination of pregnancy; P, pathogenic; LP, likely pathogenic; VUS, uncertain clinical significance; UPD, uniparental disomy; ROH, regions of homozygosity.

For the 97 cases with segmental imbalances, except for five (5.2 %) cases that were lost during follow-up, the rate of elective TOP in fetuses with clinically significant CNVs (82.9 %, 29/35) was significantly higher than that in those with variants of uncertain clinical significance (VUS) (14.0%, 8/57) (*p* < 0.001). The proportion of TOP for fetuses with VUS of *de novo* or refused parental confirmation (28.6 %, 8/30) was significantly higher than that for those with inherited VUS (0.0%, 0/33) (*p* = 0.002) (Supplementary Table S1). For the 50 cases with ROHs, except for four (8.0 %) cases that were lost to follow-up, the rate of elective TOP in fetuses with UPD (75.9 %, 22/29) was significantly higher than that in those with ROHs (29.4%, 5/17) (*p* < 0.001). There was no significant difference in the rate of elective TOP between fetuses with UPD-related imprinting disorders (100.0 %, 5/5) and those with UPD-unrelated imprinting disorders (70.8%, 17/24) (*p =* 0.222) (Supplementary Table S2). The most common reason for elective TOP in fetuses with UPD-unrelated imprinting disorders was fetal growth restriction (FGR) (29.4%, 5/17).

## Discussion

Currently, NIPT has been widely used for the detection of common fetal aneuploidies and SCAs; however, expanding the clinical applications of rare RCAs remains controversial (Rose et al., 2016; Chitty et al., 2018). According to the current guidelines (Gregg et al., 2016; American College of Obstetricians and Gynecologists’ Committee on Practice Bulletins—Obstetrics; Committee on Genetics; Society for Maternal-Fetal Medicine, 2020), NIPT is not recommended for screening RATs and genome-wide CNVs because the screening accuracy for detection and false-positive rates has not been established. The PPVs for these disorders are much lower than those for common trisomies, which may lead to unnecessary invasive procedures.

Our study showed that the PPV for RATs was low (6.1%), which is consistent with the results of previous studies (Chen et al., 2019; Zhu et al., 2021). A possible explanation for the high false-positive rate is that these RATs are less prevalent, while many of them have high rates of CPM, whereby a chromosomal abnormality occurs only in the placenta but not in the fetus, with an incidence of approximately 1%–2% in typical CVS (Grati et al., 2014; Mardy and Wapner, 2016; Grati et al., 2017; Chen et al., 2019). Notably, all the aneuploidies were confirmed to be low-level mosaicisms (13%–29%) by the SNP array of the amniotic fluid, arising from mitotic rescue of a meiotic error or a very early mitotic error (Taylor et al., 2014), which is consistent with previous studies (Liang et al., 2019; Zhu et al., 2021). The explanation for this is that almost all patients with RATs experienced pregnancy loss before amniocentesis and were excluded from our study. In addition, all parents decided to terminate the pregnancies even though no significant ultrasound abnormality was detected; this may have induced bias in comprehensively evaluating the clinical value of NIPT for RATs, especially for low-level mosaicisms without postnatal clinical features.

The PPV for segmental imbalances was moderate (21.1 %), which is consistent with the studies of Zhu et al. (2021) (28.9%) and Chen et al. (2019) (29.0%) but much lower than those reported by Liang et al. (2019) (40.8 %). The depth of sequencing may be attributable to this difference as Liang et al. (2019) performed NIPT-Plus with 20 Mb reads per sample, which was approximately four times our data. Additionally, NIPT-Plus uses combinatorial data analysis algorithms to identify genome-wide CNVs associated with MMSs (Liang et al., 2019). Comparing the results of NIPT with the SNP array, there was no significant difference among the concordance rates for subgroups of CNVs <5 Mb (75.0%), ranging from 5 to 10 Mb (86.4%), and >10 Mb (88.0%). The results oppose the empirical hypothesis that NIPT yields a higher positive rate for larger segmental imbalances than for smaller ones. This could be attributed to the optimization and validation of the regions of well-known MMSs. It is exemplified that all the four detected CNVs involving the 22q11.2 recurrent (DGS/VCFS) region were less than 3.5 Mb, which was consistent with previous studies (Ravi et al., 2018; Liang et al., 2019; Zhu et al., 2021).

For cases with clinically significant CNVs, the PPV was only 7.3% (30/413). Except for nine cases with parental chromosome rearrangements, the PPVs for clinically significant CNVs were extremely low (5.1%, 21/413). VUS accounted for 62.9 % (61/97) of the imbalanced segments; thus, the detection of VUS following positive NIPT is associated with increased family economic burden, maternal anxiety, or even panic, and the potential risk of terminating pregnancy. For the fully concordant segmental imbalances between NIPT and the SNP array, parental confirmations showed that the rate of maternal-inherited CNVs (85.3 %, 29/34) was significantly higher than that those with paternal inheritance or in a *de novo* manner (47.4 %, 9/19). Thus, it is reasonable to suspect that for gravidas with positive NIPT but negative CMA results in segmental imbalances, maternal CNVs may be detected. This could also reduce the PPVs of NIPT, as reported by Kaseniit et al. (2018) in a large-scale study. It is necessary to determine whether NIPT is an effective way to screen for clinically significant CNVs.

ROHs, termed copy number neutral segments showing continuous homozygosity with no intervening heterozygosity (Broman and Weber, 1999), were incidental findings (9.5 %) whereas the NIPT results involved aneuploidies or segmental imbalances related to the chromosomes. UPD is defined as both homologous chromosomes inherited from one parent with no contribution (for that chromosome) from the other parent (Engel, 1980). The common mechanisms resulting in UPD involve trisomy rescue, monosomy rescue, and somatic mitotic crossover (Del Gaudio et al., 2020). CPM is a well-known biologic phenomenon that is likely to result from mitotic or meiotic non-disjunction errors and trisomy rescue (Grati et al., 2014). One hypothesis is that mosaicism may be a marker for UPD (Mardy and Wapner, 2016). Although the nature process is that abnormal cell lines are encountered more frequently in placental tissues than in the fetus itself (Simoni and Sirchia, 1994), we speculated that CPM could induce abnormal NIPT results which may also be associated with UPD. The SNP array can detect isodisomy directly; however, up to one-third of UPD (heterodisomy) cases may be undetectable (Hoppman et al., 2018). Heterodisomy (exactly combined iso- and heterodisomy (mixtures of both subtypes)) may be detected by 1 or more ROHs on a single chromosome that does not include the pericentromeric region (Gonzales et al., 2022). Notably, 12 cases with combined iso- and heterodisomy were detected in our study. Thus, we recommend the prenatal SNP array for gravidas with positive NIPT results of RCAs, especially for those involved in imprinted chromosomes.

Consistent with previous studies (Wen et al., 2019; Liu et al., 2021), ROHs most frequently involved chromosomes 16 and 2, followed by chromosomes 1 and 6. After further parental confirmation, 60.0% of ROH cases were diagnosed as UPD, with a maternal to paternal ratio of 14:4, which is consistent with previous studies due to the higher propensity for maternal non-disjunction (Liehr, 2010; Yamazawa et al., 2010). In addition, five fetuses with imprinting disorders were detected. As the results of the prenatal diagnosis were obtained before detailed second-trimester fetal anomaly scans, these families opted for TOP prior to the typical ultrasound presentation of these disorders. When imprinting disorders were excluded, UPD had almost no clinical consequences (Gonzales et al., 2022). However, ROH/UPD fetuses with ultrasound abnormalities showed worse prognoses than those without abnormalities (Del Gaudio et al., 2020). In our study, 12.0% (6/50) of the cases showed FGR, one of the common ultrasound abnormalities in fetuses with ROH/UPD, which indicated adverse perinatal outcomes, and those families opted for TOP. Notably, the fetus (No. 123) with mUPD15 and fetus (No. 126) with mUPD16 were subsequently confirmed to have placental trisomy 15 and trisomy 16, respectively, which further verified the mechanism of CPM.

Our study had several limitations. First, we detected several maternal-inherited VUSs, reflecting the limitations to the analytical performance. Better algorithms to differentiate between fetal and maternal CNVs and improve clinically significant CNV calling should be performed in the future. Second, we expanded the clinical utility of NIPT for RCAs, which is recommended worldwide for traditionally screened aneuploidies. The low depth of sequencing influences the PPVs for RCAs compared with NIPT-Plus. Third, our study only included gravidas with positive NIPT results of RCAs who subsequently underwent amniocentesis for SNP analysis. We did not follow up the gravidas with negative NIPT results or refused invasive procedures. Thus, we failed to obtain a negative predictive value for comprehensively assessing the clinical use of NIPT for RCAs. Fourth, for cases with negative CMA results, we did not obtain maternal or placental results to assess the potential proportion to induce unnecessary invasive procedures. Fifth, for those fetuses with UPD/ROHs, although parental consanguinity was excluded, autosomal recessive disorders associated with ROHs were not detected regularly.

In summary, this retrospective study demonstrated that NIPT for common fetal aneuploidies yielded a low PPV for RATs (6.1 %), moderate PPV for segmental imbalances (21.1 %), and incidental findings (9.5 %) for ROH/UPD. This study provides valuable information for genetic counseling and management of gravidas with positive NIPT results for RCAs. Due to the low PPV (8.0 %) for clinically significant findings, NIPT for common fetal aneuploidies need to be noticed for rare RCAs. The prenatal SNP array should be regarded as the first-tier test for positive NIPT, particularly when imprinted chromosomes are involved.

## Data Availability

All datasets generated for this study are included in the article/supplementary material. Further inquiries can be directed to the corresponding authors.
