# Supplementary material for: Clinical experience of noninvasive prenatal testing for rare chromosome abnormalities in singleton pregnancies
Source: Front Genet. 2022 Sep 26;13:955694. doi: 10.3389/fgene.2022.955694 (PMC9549601; doi:10.3389/fgene.2022.955694)
Supplement: Supplementary file 1 [file DataSheet1.docx]

**Supplemental Table 1 Prenatal CNVs detected by CMA among the 528 gravidas with positive NIPT results**

| **No.** | **NIPT** | **CNVs (arr[GRCh37])** | **Size of CNVs (kb)** | **Copy number** | **HI/TS region** | **HI/TS gene** | **Inherited**  **or de novo** | **ACMG**  **Classification** | **Outcomes** | **Concordance** |
| --- | --- | --- | --- | --- | --- | --- | --- | --- | --- | --- |
| 1 | chr7p22.2 dup | 7p22.2(2966517_3334799)x3 | 368 | Gain | / | / | NA | VUS | Born | Full concordance |
| 2 | chr5p14.3 del | 5p14.3(19076288_19448955)x1 | 373 | Loss | / | / | NA | VUS | Born | Full concordance |
| 3 | chr13q12.11 dup | 13q12.11(22618245_22999366)x3 | 381 | Gain | / | / | NA | VUS | Born | Full concordance |
| 4 | chr3p26.2p26.1 dup | 3p26.2p26.1(3866046_4273489)x3 | 407 | Gain | / | / | Inherited from normal mother | VUS | Born | Full concordance |
| 5 | chr15q13.3 dup | 15q13.3(31999632_32428066)x3 | 428 | Gain | / | / | NA | VUS | Born | Full concordance |
| 6 | chr7q11.21 del | 7q11.21(64612880_65148399)x1 | 536 | Loss | / | / | NA | VUS | Born | Full concordance |
| 7 | chr20q13.13 del | 20q13.13(46470867_47125819)x1 | 655 | Loss | / | / | NA | VUS | Born | Full concordance |
| 8 | chr5p15.31 dup | 5p15.31(6752757_7429552)x4 | 677 | Gain | / | / | Inherited from normal mother | VUS | Born | Full concordance |
| 9 | chr7p21.1 dup | 7p21.1(17592111_18327081)x3 | 735 | Gain | / | / | Inherited from normal father | VUS | Born | Full concordance |
| 10 | chr13q12.11 dup | 13q12.11(21521989_21972234)x3 | 450 | Gain | / | / | NA | VUS | Born | Full concordance |
| 11 | chr22q11.21 del | 22q11.21(21059669_21800471)x1 | 741 | Loss | / | / | Inherited from normal mother | VUS | Born | Full concordance |
| 12 | chr22q11.21 del | 22q11.21(21059669_21800471)x1 | 741 | Loss | / | / | *de novo* | VUS | TOP | Full concordance |
| 13 | chr17q11.2 dup | 17q11.2(28749121_29516669)x3 | 768 | Gain | / | / | Inherited from normal mother | VUS | Born | Full concordance |
| 14 | chr18p11.32 dup | 18p11.32(1244515_1802917)x3 | 558 | Gain | / | / | NA | VUS | Born (Premature delivery) | Full concordance |
| 15 | chr15q13.3 del | 15q13.3(31967070_32914239)x1 | 947 | Loss | 15q13.3 recurrent region (D_CHRNA7 to BP5) (includes CHRNA7 and OTUD7A) | / | NA | P | Born | Full concordance |
| 16 | chr8q24.11 dup | 8q24.11(117990712_119012676)x3 | 1022 | Gain | / | / | Inherited from normal mother | VUS | Born | Full concordance |
| 17 | chr13q31.1 dup | 13q31.1(81924090_83110879)x3 | 1187 | Gain | / | / | Inherited from normal mother | VUS | Born | Full concordance |
| 18 | chr2p12 dup | 2p12(78631710_79851089)x4 | 1219 | Gain | / | / | NA | VUS | Born | Full concordance |
| 19 | chr13q12.12 del | 13q12.12(23533511_24970361)x1 | 1437 | Loss | / | / | Inherited from normal mother | VUS | Born | Full concordance |
| 20 | chr10q24.32q25.1 dup | 10q24.32q25.1(104583879_106039196)x3 | 1455 | Gain | / | / | NA | VUS | Born | Full concordance |
| 21 | chr13q32.1 dup | 13q32.1(95340470_96874757)x3 | 1534 | Gain | / | / | Inherited from normal mother | VUS | Born | Full concordance |
| 22 | chr2q12.2q12.3 del | 2q12.2q12.3(106856366_108527327)x1 | 1670 | Loss | / | / | NA | VUS | Born | Full concordance |
| 23 | chr16p13.12p13.11 dup | 16p13.12p13.11(14770633_16538596)x3 | 1768 | Gain | / | / | NA | VUS | Born | Full concordance |
| 24 | chr1q21.1q21.2 del | 1q21.1q21.2(146106724_147933973)x1 | 1827 | Loss | 1q21.1 recurrent region (BP3_BP4 distal) (includes GJA5) | / | Inherited from normal mother | P | Born | Full concordance |
| 25 | chr8p23.2p23.1 dup | 8p23.2p23.1(4322453_6204870)x3 | 1882 | Gain | / | / | Inherited from normal mother | VUS | Born | Full concordance |
| 26 | chr22q11.22q11.23 dup | 22q11.22q11.23(22997928_24995256)x3 | 1997 | Gain |  | / | *de novo* | VUS | Born | Full concordance |
| 27 | chr7q11.21q11.22 dup | 7q11.21q11.22(66785467_68970684)x4 | 2185 | Gain | / | / | Inherited from normal mother | VUS | Born | Full concordance |
| 28 | chr5p15.31 dup chr14q31.3 dup | 5p15.31(8827019_9798033)x3  14q31.3(85359235_87586936)x4 | 971  2228 | Gain  Gain | /  / | /  / | Inherited from normal mother  Inherited from normal mother | VUS VUS | Born | Full concordance |
| 29 | chr16q21 dup | 16q21(61996041_64301745)x3 | 2306 | Gain | / | / | Inherited from normal mother | VUS | Born | Full concordance |
| 30 | chr22q11.21 del | 22q11.21(18648855_21062134)x1 | 2413 | Loss | 22q11.2 recurrent (DGS/VCFS) region (proximal A_B) (includes TBX1) | / | *de novo* | P | TOP | Full concordance |
| 31 | chr18q22.1 dup | 18q22.1(64283071_66769260)x3 | 2486 | Gain | / | / | NA | VUS | NA | Full concordance |
| 32 | chr21q dup | 21q22.3(43941791_46523623)x3 | 2582 | Gain | / | / | Inherited from normal mother | VUS | Born | Full concordance |
| 33 | chr22q11.21 dup | 22q11.21(18627051_21283290)x3 | 2656 | Gain | 22q11.2 recurrent (DGS/VCFS) region (proximal A_B) (includes TBX1) | / | NA | P | TOP | Full concordance |
| 34 | chr12q24.32q24.33 del | 12q24.32q24.33(127391909_130111679)x1 | 2720 | Loss | / | / | Inherited from normal father | VUS | Born (Premature delivery) | Full concordance |
| 35 | chr14q21.2q21.3 del | 14q21.2q21.3(46210321_49016299)x1 | 2806 | Loss | / | / | Inherited from normal mother | VUS | Born (hydronephrosis) | Full concordance |
| 36 | chr22q11.21 dup | 22q11.21(18648856_21461017)x3 | 2812 | Gain | 22q11.2 recurrent (DGS/VCFS) region (proximal A_B) (includes TBX1) | / | NA | P | TOP | Full concordance |
| 37 | chr16p13.11p12.3 dup | 16p13.11p12.3(15325073_18157612)x3 | 2833 | Gain | / | / | NA | VUS | Born | Full concordance |
| 38 | chr22q11.21 del | 22q11.21(18631364_21800471)x1 | 3169 | Loss | 22q11.2 recurrent (DGS/VCFS) region (proximal A_D) (includes TBX1) | / | NA | P | TOP | Full concordance |
| 39 | chr6p12.3 dup | 6p12.3(46466383_50480392)x3 | 4014 | Gain | / | / | Inherited from normal mother | VUS | Born | Full concordance |
| 40 | chr2q12.2q13 del | 2q12.2q13(107040325_111370025)x1 | 4330 | Loss | / | / | Inherited from normal mother | VUS | Neonatal death | Full concordance |
| 41 | chr16q11.2q12.1 dup | 16q11.2q12.1(46503969_51098261)x3 | 4594 | Gain | / | / | NA | VUS | Born | Full concordance |
| 42 | chr21q21.1 dup | 21q21.1(18490110_23347274)x3 | 4857 | Gain | / | / | Inherited from normal mother | VUS | Born | Full concordance |
| 43 | chr15q11.2q13.1 dup | 15q11.2q13.1(23632678_28560664)x3 | 4928 | Gain | 15q11q13 recurrent (PWS/AS) region (BP2_BP3 Class 2) | / | Inherited from normal mother | P | Born | Full concordance |
| 44 | chr5q22.1q23.1 dup | 5q22.1q23.1(110896866_116195651)x3 | 5299 | Gain | / | / | NA | VUS | TOP | Full concordance |
| 45 | chr8q23.1q23.3 dup | 8q23.1q23.3(110273153_115684011)x3 | 5411 | Gain | / | / | NA | VUS | Born | Full concordance |
| 46 | chr11p15.1p14.3 dup | 11p15.1p14.3(20253705_25684613)x3 | 5431 | Gain | / | / | Inherited from normal mother | VUS | Born | Full concordance |
| 47 | chr11p15.1p14.3 dup | 11p15.1p14.3(20277669_25713381)x3 | 5436 | Gain | / | / | Inherited from normal mother | VUS | Born | Full concordance |
| 48 | chr10q11.22q11.23 del | 10q11.22q11.23(46293590_51817663)x1 | 5524 | Loss | / | / | Inherited from normal mother | VUS | Born | Full concordance |
| 49 | chr15q26.1q26.3 dup | 15q26.1q26.3(93404310_98968661)x3 | 5564 | Gain | / | / | NA | VUS | Born | Full concordance |
| 50 | chr16p13.13p12.3 del | 16p13.13p12.3(12548052_18242713)x1 | 5695 | Loss | 16p13.11 recurrent region (BP2_BP3) (includes MYH11) | / | NA | P | Born | Full concordance |
| 51 | chr15q11.2q13.1 dup | 15q11.2q13.1(22770421_28526905)x3 | 5756 | Gain | 15q11q13 recurrent (PWS/AS) region (BP1_BP3 Class 1) | / | Inherited from normal mother | P | NA | Full concordance |
| 52 | chr12q21.2q21.31 del | 12q21.2q21.31(79313475_85441579)x1 | 6128 | Loss | / | PPP1R12A | NA | P | Born | Full concordance |
| 53 | chr8q21.13q21.3 del | 8q21.13q21.3(80275606_87340145)x1 | 7065 | Loss | / | / | NA | VUS | TOP | Full concordance |
| 54 | chr10q22.3q23.2 dup | 10q22.3q23.2(81674867_88957815)x3 | 7283 | Gain | / | / | Inherited from normal mother | VUS | Born | Full concordance |
| 55 | chr10q22.3q23.2 del | 10q22.3q23.2(81630469_88957815)x1 | 7327 | Loss | 10q22.3q23.2 recurrent region (LCR_3/4_flanked) (includes BMPR1A) | BMPR1A/PTEN | NA | P | TOP | Full concordance |
| 56 | chr10q22.3q23.2 dup | 10q22.3q23.2(81630469_88973570)x3 | 7343 | Gain | / | / | Inherited from normal mother | VUS | Born | Full concordance |
| 57 | chr7p21.3p21.1 del | 7p21.3p21.1(8974812_17191226)x1 | 8216 | Loss | / | / | *de novo* | VUS | TOP | Full concordance |
| 58 | chr11q24.2q25 del | 11q24.2q25(126647772_134937416)x1 | 8290 | Loss | / | / | *de novo* | VUS | TOP | Full concordance |
| 59 | chr3q11.1q12.3 dup | 3q11.1q12.3(93519465_101839691)x3 | 8320 | Gain | / | / | Inherited from normal mother | VUS | NA | Full concordance |
| 60 | chr8p23.3p23.1 del | 8p23.3p23.1(158048_9010029)x1 | 8852 | Loss | / | / | NA | LP | TOP | Full concordance |
| 61 | chr5q21.1q22.1 dup | 5q21.1q22.1(101522649_110634622)x3 | 9112 | Gain | / | / | Inherited from normal mother | VUS | Born | Full concordance |
| 62 | chr5p11q11.2 dup | 5p11q11.2(46365514_55986750)x3 | 9621 | Gain | / | / | Inherited from normal mother | VUS | Born | Full concordance |
| 63 | chr2p23.1p22.1 del | 2p23.1p22.1(30733152_41085497)x1 | 10352 | Loss | / | SPAST | NA | P | Born | Full concordance |
| 64 | chr8p23.3p23.1 del | 8p23.3p23.1(158049_10685851)x1 | 10528 | Loss | / | / | NA | LP | TOP | Full concordance |
| 65 | chr3q27.2q29 dup | 3q27.2q29(185409997_197851444)x3 | 12441 | Gain | / | / | NA | LP | TOP | Full concordance |
| 66 | chr5q21.3q23.1 del | 5q21.3q23.1(104832795_118713574)x1 | 13881 | Loss | / | APC | NA | P | TOP | Full concordance |
| 67 | chr21q11.2q21.3 del | 21q11.2q21.3(15016487_29188153)x1 | 14172 | Loss | / | / | *de novo* | VUS | NA | Full concordance |
| 68 | chr4q13.1q21.1 del | 4q13.1q21.1(62323426_76675789)x1 | 14352 | Loss | / | / | *de novo* | P | TOP | Full concordance |
| 69 | chr18q22.1q23 del | 18q22.1q23(63578361_78013728)x1 | 14435 | Loss | / | EYA1 | Inherited from mother with intellectual disability | P | NA | Full concordance |
| 70 | chr18p11.32 del  chr18p11.32p11.21 dup | 18p11.32(136228_1331930)x1  18p11.32p11.21(1343954_15170636)x3 | 1196  13827 | Loss  Gain | /  / | /  / | NA | VUS LP | Born | Full concordance |
| 71 | chr18p11.32q23 dup | 18p11.32q23(136228_15181207).15 | 15045 | Gain (mosaic) | / | / | NA | LP | TOP | Full concordance |
| 72 | chr2q23.3q31.1 dup | 2q23.3q31.1(154358188_170720261)x3 | 16362 | Gain | / | / | NA | LP | TOP | Full concordance |
| 73 | chr5p15.33p14.3 del | 5p15.33p14.3(113576_18727376)x1 | 18614 | Loss | 5p15 terminal (Cri du chat syndrome) region | TRIO | NA | P | TOP | Full concordance |
| 74 | chr4q31.3q34.2 del | 4q31.3q34.2(154851387_176868942)x1 | 22018 | Loss | / | / | NA | P | TOP | Full concordance |
| 75 | chr5p15.33p14.1 del | 5p15.33p14.1(113577_26243789)x1 | 26130 | Loss | 5p15 terminal (Cri du chat syndrome) region | TRIO | NA | P | TOP | Full concordance |
| 76 | chr18p11.22 dup | 18p11.22(8718383_10139732)x3  22q11.23q12.1(25116001_26437690)x3 | 1421  1322 | Gain  Gain | /  / | /  / | Inherited from normal mother  *de novo* | VUS VUS | TOP (hydrocephalus) | Partial concordance |
| 77 | chr8p23.3p23.2 del | 8p23.3p23.2(158048_3220759)x1 8q21.11q24.3(77115706_146295771)x3 | 3063  69180 | Loss  Gain | /  / | /  / | Paternal invertion: 46,XY,inv(8)(p23.2;q21) ***** | VUS LP | TOP | Partial concordance |
| 78 | chr8p23.3p23.2 del | 8p23.3p23.2(158048_4745371)x1 9p24.3p21.3(208454_22492876)x3 | 4587  22284 | Loss  Gain | /  / | /  / | Maternal balanced  translocation: 46,XX,t(8;9)(p23;p21.3) | VUS LP | TOP | Partial concordance |
| 79 | chr20p13p12.1 dup | 20p13p12.1(61662_13546848)x3  9p24.3p23(208455_11144684)x1 | 13426  10936 | Gain  Loss | /  / | /  / | Paternal balanced  translocation: 46,XY,t(9;20)(p23;p12.1) | LP LP | TOP | Partial concordance |
| 80 | chr9p24.3p22.3 dup | 9p24.3p22.3(208455_15608372)x3  5p15.33(113577_1641914)x1 | 15400  1528 | Gain  Loss | /  / | /  / | Inherited from mother with intellectual disability: 46,XX,der t(5;9)(p15.3;p22) | VUS VUS | TOP | Partial concordance |
| 81 | chr12q24.21q24.33 dup | 12q24.21q24.33(116785882_133777562)x3  2q37.3(240495629_242782258)x1 | 16992  2287 | Gain  Loss | /  / | /  / | Maternal balanced  translocation: 46,XX,t(2;12)(q37;q24) | LP VUS | TOP | Partial concordance |
| 82 | chr18q21.33q23 dup | 18q21.33q23(60906988_78013728)x3  4q35.2(187316147_190957460)x1 | 17107  3641 | Gain  Loss | /  / | /  / | Inherited from mother with intellectual disability: 46,XX,der t(4;18)(q35;q22) | VUS VUS | TOP | Partial concordance |
| 83 | chr17q23.3q25.3 dup | 17q23.3q25.3(61775472_81041823)x3 1p36.33p36.32(849466_3777765)x1 | 19266 2928 | Gain Loss | / 1p36 terminal region (includes GABRD) | /  / | Maternal balanced  translocation: 46,XX,t(1;17)(p36;q23) | LP P | TOP | Partial concordance |
| 84 | chr18q21.32q23 del | 18q21.32q23(58106726_78013728)x1  1q43(238949246_240062389)x1 | 19907  1113 | Loss  Loss | /  / | /  / | NA | LP VUS | TOP | Partial concordance |
| 85 | chr9p24.3p13.2 dup | 9p24.3p13.2(1581286_37055141)x3  9p24.3(208454_1577575)x1 | 35474  1369 | Gain  Loss | /  / | /  / | NA | LP VUS | TOP | Partial concordance |
| 86 | chr20p13p11.21 dup | 20p13p11.21(61661_24487341)x3  11q24.2q25(126392021_134937416)x1 | 24426  8545 | Gain  Loss | /  / | /  / | Maternal balanced  translocation: 46,XX,t(11;20)(q24.2;p11.2) | LP VUS | TOP | Partial concordance |
| 87 | chr3p26.3p22.2 dup | 3p26.3p22.2(285857_37597219)x3 5p15.33p15.31(113577_8750244)x1 | 37311  8637 | Gain  Loss | / 5p15 terminal (Cri du chat syndrome) region | /  TRIO | Paternal balanced  translocation: 46,XY,t(3;5)(p24;p15.3) | VUS P | TOP | Partial concordance |
| 88 | chr14q del | Yq11.21q11.221(14460771_15220682)x0 | 760 | Loss | / | / | NA | VUS | Born | Discordance |
| 89 | chr7q del | 16p11.2(29428531_30190029)x1 | 761 | Loss | 16p11.2 recurrent region (proximal BP4_BP5) (includes TBX6) | / | *de novo* | P | TOP | Discordance |
| 90 | chr1p del chr4q dup | 4q35.2(190114475_190957460)x1 | 843 | Loss | / | / | NA | VUS | Born | Discordance |
| 91 | chr15q dup | 10p13p12.33(17066844_18286639)x3 | 1220 | Gain | / | / | Inherited from normal mother | VUS | Born | Discordance |
| 92 | chr7q11.22 del | Xp22.31(6449837_8143509)x1 | 1694 | Loss | Xp22.31 recurrent region (includes STS) | STS | NA | P | Born | Discordance |
| 93 | chr18p11.23 dup | 4q34.3(178130291_179860825)x3 | 1731 | Gain | / | / | NA | VUS | Born | Discordance |
| 94 | chr5q15 dup | 8q24.12(119740530_122337637)x3 | 2597 | Gain | / | / | Inherited from normal father | VUS | Born | Discordance |
| 95 | chr11p13 del | 1q25.3q31.1(184825946_187563410)x1 | 2737 | Loss | / | / | Inherited from normal mother | VUS | Born | Discordance |
| 96 | chr14q24.1 dup | 5p15.1p14.3(17897900_21148212)x3 | 3250 | Gain | / | / | Inherited from normal mother | VUS | Born | Discordance |
| 97 | chr14q24.3 del | 5p15.33p15.32(113577_6138632)x1 | 6025 | Loss | 5p15 terminal (Cri du chat syndrome) region | TRIO | NA | P | TOP | Discordance |

CNV: copy number variation; CMA: chromosomal microarray analysis; NIPT: noninvasive prenatal testing; HI: haploinsufficiency; TS: triplosensitivity; ACMG: American College of Medical Genetics and Genomics; NA: not available; P: pathogenic; LP: likely pathogenic; VUS: uncertain clinical significance; TOP: termination of pregnancy

**Supplemental Table 2 Prenatal ROHs detected by CMA among the 528 gravidas with positive NIPT results**

| **No.** | **NIPT** | **CNVs (arr[GRCh37])** | **Size of ROHs (kb)** | **Copy number** | **Disorder** | **Source** | **Outcomes** |
| --- | --- | --- | --- | --- | --- | --- | --- |
| 98 | trisomy 1 | 1p36.33p11.2(888658_121339317)x2 hmz  1q21.2q44(149879544_249198164)x2 hmz | 120451  99319 | UPD1 (isodisomy) | / | NA | TOP |
| 99 | trisomy 1 | 1p36.33p11.2(888658_121339317)x2 hmz  1q21.2q44(149879544_249198164)x2 hmz | 120451  99319 | UPD1 (isodisomy) | / | NA | TOP (FGR) |
| 100 | trisomy 1 | 1p36.33p11.2(888658_121339317)x2 hmz  1q21.2q44(149879544_249198164)x2 hmz | 120451  99319 | UPD1 (isodisomy) | / | NA | Born (Methylmalonic acidemia) |
| 101 | trisomy 2 | 2p25.3p11.2(50813_87053152)x2 hmz  2q11.1q37.3(95550957_242773583)x2 hmz | 87002  147223 | UPD2 (isodisomy) | / | NA | TOP (FGR) |
| 102 | trisomy 2 | 2p25.3p11.2(50813_87053152)x2 hmz  2q11.1q37.3(95550957_242773583)x2 hmz | 87002  147223 | mUPD2 (isodisomy) | / | maternal | TOP (hydrops fetalis) |
| 103 | trisomy 2 | 2p24.1p16.1(19693805_59685825)x2 hmz  2q14.2q24.1(121278207_158756848)x2 hmz  2q31.1q34(175042562_213345197)x2 hmz | 39992  37479  38303 | mUPD2 (iso_heterodisomy*) | / | maternal | Born |
| 104 | trisomy 3 | 3p26.3p25.1(73602_16294894)x2 hmz  3p12.3p11.1(74601403_88586090)x2 hmz  3q11.1q13.11(93558925_105429152)x2 hmz | 16221  13985  11870 | mUPD3 (iso_heterodisomy) | / | maternal | NA |
| 105 | trisomy 4 | 4p16.3p11(75173_49063479)x2 hmz  4q11q35.2(52696791_190921709)x2 hmz | 48988  138225 | UPD4 (isodisomy) | / | NA | TOP |
| 106 | trisomy 4 | 4p16.3p11(75173_49063479)x2 hmz  4q11q35.2(52696791_190921709)x2 hmz | 48988  138225 | pUPD4 (isodisomy) | / | paternal | TOP (FGR) |
| 107 | trisomy 4 | 4p16.3p11(75173_49063479)x2 hmz  4q11q35.2(52696791_190921709)x2 hmz | 48988  138225 | UPD4 (isodisomy) | / | NA | Born |
| 108 | trisomy 4 | 4p16.3p15.33(75173_15121280)x2 hmz  4p14p11(38705256_49063479)x2 hmz  4q28.2q34.2(129685157_177412472)x2 hmz | 15046  10358  47727 | mUPD4 (iso_heterodisomy) | / | maternal | TOP |
| 109 | trisomy 6 | 6p25.3p11.1(203877_58726706)x2 hmz  6q11.1q27(61972917_170896644)x2 hmz | 58523  108924 | pUPD6 (isodisomy) | Transient Neonatal  Diabetes mellitus (TNDM) | paternal | TOP (FGR) |
| 110 | trisomy 6 | 6p25.3p11.1(203877_58726706)x2 hmz  6q11.1q27(61972917_170896644)x2 hmz | 58523  108924 | mUPD6 (isodisomy) | / | maternal | Born |
| 111 | trisomy 6 | 6p25.3p11.1(203877_58726706)x2 hmz  6q11.1q27(61972917_170896644)x2 hmz | 58523  108924 | UPD6 (isodisomy) | / | NA | TOP |
| 112 | trisomy 6 | 6p24.1p11.1(13395610_58726706)x2 hmz  6q16.1q22.31(94446431_124307093)x2 hmz  6q25.1q27(150710446_170896644)x2 hmz | 45331  29861  20186 | mUPD6 (iso_heterodisomy) | / | maternal | TOP (FGR) |
| 113 | trisomy 6 | 6p25.3p11.1(203878_58726706)x2 hmz 6q11.1q27(61972918_170896644)x2 hmz | 58523  108924 | mUPD6 (isodisomy) | / | maternal | Born |
| 114 | trisomy 7 | 7p22.3p11.1(50943_58019983)x2 hmz  7q11.21q36.3(62569501_159118443)x2 hmz | 57969  96549 | UPD7 (isodisomy) | / | NA | Born (short stature) |
| 115 | trisomy 7 | 7p22.3p11.1(50943_58019983)x2 hmz  7q11.21q36.3(62569501_159118443)x2 hmz | 57969  96549 | UPD7 (isodisomy) | / | NA | TOP |
| 116 | trisomy 8 | 8p23.1p11.1(8117564_43776564)x2 hmz  8q11.1q24.3(46919156_146292734)x2 hmz | 35659  99374 | mUPD8 (isodisomy) | / | maternal | TOP |
| 117 | trisomy 8 | 8p23.1p11.1(8117564_43776564)x2 hmz  8q11.1q24.3(46919156_146292734)x2 hmz | 35659  99374 | UPD8 (isodisomy) | / | NA | TOP |
| 118 | trisomy 9 | 9p24.3p13.1(216123_38771831)x2 hmz 9q21.11q34.3(71013799_141011581)x2 hmz | 38556  69998 | UPD9 (isodisomy) | / | NA | TOP |
| 119 | trisomy 9 | 9p24.3p13.1(216124_38771831)x2 hmz  9q21.11q34.3(71013800_141011581)x2 hmz | 38556  69998 | pUPD9 (iso_heterodisomy) | / | paternal | TOP |
| 120 | trisomy 11 | 11q23.2q25(112793095_134930689)x2 hmz | 22138 | mUPD11 (iso_heterodisomy) | Silver–Russell  syndrome (SRS) | maternal | TOP |
| 121 | trisomy 11 | 11q14.2q25(88151706_134930689)x2 hmz | 46779 | pUPD11 (iso_heterodisomy) | Beckwith–Wiedemann  syndrome (BWS) | paternal | TOP |
| 122 | chr14q23.3q24.3 dup dup | 14q23.1q32.11(61630461_91714413)x2 hmz | 30084 | mUPD14 (iso_heterodisomy) | Temple syndrome | maternal | TOP |
| 123 | chr15q21.2 dup | 15q21.1q22.2(48078591_62709924)x2 hmz | 14631 | mUPD15 (iso_heterodisomy) | Prader–Willi  syndrome (PWS) | maternal | TOP  Placental trisomy 15 confirmed by FISH |
| 124 | trisomy 16 | 16p13.3p13.13(94808_11870494)x2 hmz  16q23.1q24.3(77786018_90146366)x2 hmz | 11776  12360 | mUPD16 (iso_heterodisomy) | / | maternal | TOP |
| 125 | trisomy 16 | 16p13.3p12.3(94807_20050658)x2 hmz  16q22.1q24.3(69860932_90146366)x2 hmz | 19956  20285 | mUPD16 (iso_heterodisomy) | / | maternal | Born |
| 126 | trisomy 16 | 16p13.3p13.13(94807_11219041)x2 hmz  16q22.3q24.3(73469057_90146366)x2 hmz | 11124  11667 | mUPD16 (iso_heterodisomy) | / | maternal | TOP (demise)  Placental trisomy 16 confirmed by FISH |
| 127 | trisomy 17 | 17p13.3p11.2(18900_22170994)x2 hmz  17q11.1q25.3(25309336_81041760)x2 hmz | 22152  55732 | UPD17 (isodisomy) | / | NA | TOP (FGR) |
| 128 | trisomy 1 | 1p13.3p11.2(108503808_121339317)x2 hmz  1q21.2q24.3(149879544_172597553)x2 hmz  1q41q43(218237293_241346599)x2 hmz | 12836  22718  23109 | ROH | / | NA | Born |
| 129 | trisomy 1 | 1p32.2p21.3(58259167_99399687)x2 hmz  1q25.3q42.12(182520611_226528744)x2 hmz | 41141  44008 | ROH | / | NA | NA |
| 130 | trisomy 1 | 1p13.2p11.2(115835500_121339317)x2 hmz  1q21.2q23.3(149879545_165480347)x2 hmz | 5504  15601 | ROH | / | NA | Born |
| 131 | trisomy 2 | 2p25.3p22.2(1989288_37998488)x2 hmz  2q36.1q37.3(223571444_242773583)x2 hmz | 36009  19202 | ROH | / | NA | Born (short stature) |
| 132 | trisomy 2 | 2p13.2p11.2(72382277_87053152)x2 hmz  2q11.1q12.3(95550957_109616111)x2 hmz | 14671  14065 | ROH | / | NA | Neonatal death |
| 133 | trisomy 2 | 2p12p11.2(75698714_87053152)x2 hmz  2q11.1q12.2(95550957_106174659)x2 hmz | 11354  10624 | ROH | / | NA | Born |
| 134 | trisomy 3 | 3p26.3p26.1(73603_6891874)x2 hmz 3p22.2p13(39251141_69830674)x2 hmz  3q25.1q27.2(150678233_185795060)x2 hmz | 6818  30580  35117 | ROH | / | NA | TOP |
| 135 | trisomy 6 | 6p25.3p11.1(203877_58726706)x2 hmz | 58523 | ROH | / | NA | TOP (LVOTO) |
| 136 | trisomy 8 | 8p12p11.1(31079982_43776564)x2 hmz  8q11.1q12.2(46919156_61854841)x2 hmz | 12697  14936 | ROH | / | NA | Born |
| 137 | trisomy 8 | 8p21.3p11.23(20832793_36638339)x2 hmz  8q12.3q22.1(65927736_93643205)x2 hmz | 15806  27715 | ROH | / | NA | NA |
| 138 | trisomy 8 | 8p12p11.1(34070872_43776564)x2 hmz  8q11.1q13.2(46919157_68771501)x2 hmz | 97057  21852 | ROH | / | NA | Born |
| 139 | trisomy 9 | 9p24.3p21.3(216123_20570700)x2 hmz  9q31.3q34.3(112814078_141011581)x2 hmz | 20355  28198 | ROH | / | NA | TOP |
| 140 | trisomy 12 | 12p13.33p11.22(257935_30396571)x2 hmz | 30139 | ROH | / | NA | Born |
| 141 | trisomy 13 | 13q31.1q34(84417821_115095705)x2 hmz | 30678 | ROH | / | NA | Born |
| 142 | trisomy 16 | 16p13.3p13.13(94808_12292798)x2 hmz 16q23.2q24.3(80394565_90146366)x2 hmz | 12198  9752 | ROH | / | NA | NA |
| 143 | trisomy 16 | 16q23.1q24.3(78950254_90146366)x2 hmz | 11196 | ROH | / | NA | Born |
| 144 | trisomy 16 | 16p13.3p13.12(94808_14053831)x2 hmz | 13959 | ROH | / | NA | TOP |
| 145 | trisomy 16 | 16p13.3p12.3(94808_19331243)x2 hmz | 19236 | ROH | / | NA | Born |
| 146 | trisomy 16 | 16p13.3(94808_7154181)x2 hmz  16q23.3q24.3(81940867_90146366)x2 hmz | 7059  8206 | ROH | / | NA | Born |
| 147 | trisomy 16 | 16p13.3(94808_5500174)x2 hmz  16q21q23.3(66159040_83117017)x2 hmz | 5405  16958 | ROH | / | NA | fetal loss after  amniocentesis |

CMA: chromosomal microarray analysis; NIPT: noninvasive prenatal testing; UPD:uniparental disomy; ROH: regions of homozygosity; NA: not available; TOP: termination of pregnancy; FGR: fetal growth restriction; LVOTO: left ventricular outflow tract; FISH: fluorescence in situ hybridization

*: Iso_heterodisomy indicates combined isodisomy and heterodisomy (mixtures of both subtypes).
